# Isolation and molecular characterization of *Mannheimia haemolytica* and *Pasteurella multocida* associated with pneumonia of goats in Chhattisgarh

**DOI:** 10.14202/vetworld.2019.331-336

**Published:** 2019-02-24

**Authors:** Nidhi Rawat, Varsha Rani Gilhare, Krishna Kumar Kushwaha, Deeksha Dipak Hattimare, Foziya Farzeen Khan, Rajesh Kumar Shende, Dhananjay Kumar Jolhe

**Affiliations:** 1 Department of Veterinary Microbiology, College of Veterinary Science and Animal Husbandry, Anjora, Durg, Chhattisgarh, India; 2 Department of Veterinary Public Health and Epidemiology, College of Veterinary Science and Animal Husbandry, Anjora, Durg, Chhattisgarh, India; 3 Department of Veterinary Pathology, College of Veterinary Science and Animal Husbandry, Anjora, Durg, Chhattisgarh, India

**Keywords:** bronchopneumonia, goat, *Mannheimia haemolytica*, *Pasteurella multocida*, pneumonic pasteurellosis

## Abstract

**Aim:**

The purpose of this study was to isolate and characterize the *Mannheimia haemolytica* and *Pasteurella multocida* from blood, nasal discharge, and lung tissue of pneumonic goats.

**Materials and Methods:**

A total of 14 goats were investigated for pneumonic pasteurellosis. Of 14 goats, nasal swabs and blood samples were collected from 10 clinically diseased animals. Moreover, lung tissue and heart blood samples were collected during necropsy of four goats died with pneumonia. All the samples were processed for the isolation of *M. haemolytica* and *P. multocida* in the laboratory. Bacterial isolates were identified by cultural and biochemical characters and 16S rRNA sequence analysis. All the isolates were subjected to susceptibility testing using commonly used antimicrobials. *M. haemolytica* isolates were characterized by *PHSSA* gene detection. *P. multocida* isolates were characterized by *KMT1* gene detection and capsule typing.

**Results:**

On necropsy of dead goats, the pneumonia was characterized as acute fibrinous bronchopneumonia. Bacterial culture revealed the isolation of *M. haemolytica* (7) and *P. multocida* (5) of 10 clinical cases. Moreover, *M. haemolytica* and *P. multocida* were coisolated from two of the lung tissues. Furthermore, one of the other two lung tissues showed the isolation of *M. haemolytica* while the other showed recovery of *P. multocida*. Bacterial isolates were specifically identified by the 16S rRNA sequence analysis. The isolates showed reduced susceptibility to β-lactams, aminoglycosides, and fluoroquinolones. Moreover, the *PHSSA* and *KMT1* genes were specifically detected among *M. haemolytica*, and *P. multocida* isolates, respectively. All *P. multocida* isolates belonged to serogroup A.

**Conclusion:**

The present study reported an occurrence of pneumonic pasteurellosis caused by *M. haemolytica* and *P. multocida* in a goat flock.

## Introduction

Pneumonic pasteurellosis, also known as respiratory mannheimiosis, is most common among the respiratory tract infections with a wide prevalence in ruminant animals. Small ruminants such as goats are fairly susceptible and contract the disease due to exposure to physical stress or unfavorable environmental conditions [1]. It is one of the most common respiratory illnesses in goats throughout the world [2]. Pneumonia in goat is clinically characterized by anorexia, fever (40-41°C), painful coughing, dyspnea, mucopurulent nasal discharge, and depression.

Bacterial pneumonia is most often caused by *Mannheimia haemolytica* and *Pasteurella multocida* which are more frequently associated with the outbreak of acute pneumonia and death of goats in all age groups [3]. *P. multocida*, an opportunistic pathogen, is analogous to *M. haemolytica* [4] and is one of the most important respiratory pathogens of domestic ruminants in which it causes serious outbreaks of acute pneumonia [2,5]. This syndrome is caused by a complex interaction between the environmental stress, microorganisms, and immunity of the host. Although pneumonia in small ruminants is primarily caused by certain viral agents and is predisposed by the extremes of environmental insults, *M. haemolytica* is the most frequently detected bacterial pathogen [6]. Furthermore, *M. haemolytica* promotes *P. multocida* colonization in lung tissue, leading to more severe disease [7].

Presumptive bacterial identification may be made on the basis of cultural and biochemical features. However, molecular methods are required for confirmatory bacterial identification [8]. The goat is an important food animal for sustaining the livelihood of various rural families in Chhattisgarh, India. Therefore, it was planned to isolate and characterize *M. haemolytica* and *P. multocida* associated with clinical pneumonia in goats.

## Materials and Methods

### Ethical approval

The approval from the Institutional Animal Ethics Committee was not required for the present study since the samples were collected from the animals without animal experimentation and the dead animals during the necropsy.

### Collection and processing of samples

A total of 14 goats (six adults, two hogget, and six suckling kids) in a backyard flock of 25 goats, maintained in one of the villages of Dongargaon block of district Rajnandgaon in the state of Chhattisgarh, were clinically ill and showing anorexia, fever, mucoid nasal discharge, and coughing. Moreover, four goats including one adult and three suckling kids suddenly died after an episode of fever and respiratory signs. All the animals were investigated for the occurrence of pneumonic pasteurellosis. The nasal swabs were collected from all the diseased animals (n=10) showing respiratory symptoms. Lung tissue samples were collected from dead animals (n=04) at the time of necropsy. For sampling during necropsy of the dead animals, the outer surface of the pneumonic lungs was first cleaned by touching the lung surface with a heated spatula before cutting their inner surface. The inner part of lung tissue from each animal was collected with aseptic measures for the isolation of *M. haemolytica* and *P. multocida*. Simultaneously, the heart blood samples were collected directly in the sterile syringes. The lung tissue samples were aseptically processed by making 20% w/v tissue homogenate using sterilized phosphate buffer saline (PBS) in laminar air flow cabinet near flame. The nasal swabs were suspended in 0.5 ml of the sterilized PBS.

### Bacterial isolation and identification

All the samples were directly streaked on blood agar base (HiMedia, Mumbai) supplemented with 7% defibrinated goat blood and incubated aerobically for 24-48 h at 37°C. The plates were then examined for bacterial growth and the colonies examined for colony morphology, color, and odor. The suspected colonies were stained by Gram staining, observed, and tested for the following characteristics: presence or absence of hemolysis on blood agar, motility, indole and oxidase production, and growth on MacConkey agar (HiMedia, Mumbai). Gram-negative coccobacilli identified by the standard cultural and biochemical tests above were selected for further identification of *P. multocida* or *M. haemolytica* [9]. The pure bacterial colonies were maintained in brain heart infusion (BHI) agar (HiMedia, Mumbai) slants for further use.

### Antimicrobial susceptibility testing

All of *P. multocida* and *M. haemolytica* isolates were tested for their susceptibility to amoxicillin (30 µg), cefotaxime (30 µg), amikacin (30 µg), gentamicin (10 µg), ciprofloxacin (5 µg), enrofloxacin (5 µg), tetracycline (30 µg), and chlortetracycline (30 µg) disks (HiMedia, Mumbai) using disk diffusion method [10]. *Escherichia coli* ATCC 25922 was used as a quality control strain. The interpretation of the results was based on the Clinical and Laboratory Standards Institute breakpoints [11].

### Isolation of DNA from bacterial colonies

The isolates presumptively identified as *M. haemolytica* and *P. multocida* were streaked on BHI agar (HiMedia, Mumbai) and incubated aerobically at 37°C for 48 h to isolate the genomic DNA. About 5-10 colonies of each of the pure isolates of *M. haemolytica* and *P. multocida* were transferred into 1.5 ml Eppendorf tubes. The bacterial colonies were washed twice in nuclease-free water by centrifugation at 10,000 rpm for 3 min in a microcentrifuge. The genomic DNA was isolated using DNeasy Blood and Tissue Kit (Qiagen, USA) as per the manufacturer’s instructions.

### Polymerase chain reaction (PCR) amplification

PCR amplification was carried out using the specific primers (Sigma-Aldrich, USA) to identify the bacterial isolates (Table-1) [12-15]. The reaction mixtures and amplification conditions were optimized for all the genes. *M. haemolytica* and *P. multocida* isolates were specifically identified based on the amplification of 16S rRNA using universal primers. Further, *M. haemolytica* and *P. multocida* were identified by species-specific amplification of the *PHSSA* and *KMT1* gene, respectively. *P. multocida* isolates were subjected to capsule typing. For PCR amplification, 50 ng of DNA was added to 25 µl reaction mixture containing 200 µM of dNTPs, 0.2 µM of each primer, 1.875 mM of MgCl_2_, and 1.25 U of Taq DNA polymerase (Sigma-Aldrich, USA) in 1×PCR buffer. PCR conditions for *PHSSA* included the initial denaturation at 95°C for 3 min, followed by 35 cycles consisted of denaturation at 95°C for 1 min, annealing at 48°C for 1 min, extension at 72°C for 30 s, and the final extension at 72°C for 5 min. PCR conditions for the *KMT1* gene comprised initial denaturation at 95°C for 3 min, followed by 35 cycles consisted of denaturation at 95°C for 45 s, annealing at 56°C for 45 s, extension at 72°C for 1 min, and the final extension at 72°C for 5 min. The amplified PCR products (5 µl) were separated in agarose gel (1.5% w/v) stained with ethidium bromide (0.5 µg/ml) by running in horizontal submarine electrophoresis unit using 1×TAE as running buffer and examined under the gel documentation system (UVP, USA).

**Table-1:** Primer sequence of different genes.

Gene	Primer sequence	Amplicon size (bp)	Annealing temp (°C)	References
*16S rRNA*	27F 5`AGAGTTTGATCMTGGCTCAG3`	~1466	52	[12]
	1492R 5`CGGTTACCTTGTTACGACTT3`			
*PHSSA*	F 5` TTCACATCTTCATCCTC3`	327	48	[13]
	R 5` TTTTCATCCTCTTCGTC3			
*KMT1*	F 5`ATCCGCTATTTACCCAGTGG3`	457	56	[14]
	R 5`GCTGTAAACGAACTCGCCAC3`			
*hyaD-hyaC*	F 5`TGCCAAAATCGCAGTCAG3`	1046	55	[15]
	R 5`TTGCCATCATTGTCAGTG3			

### Nucleotide sequencing

The amplified PCR products (50 µl) were purified using gel extraction kit (Qiagen, USA) as per the manufacturer’s instructions. The purified PCR products were quantified (~100 ng/µl concentration) and used for sequencing by bidirectional Sanger`s sequencing method.

## Results

**Figure-1:**
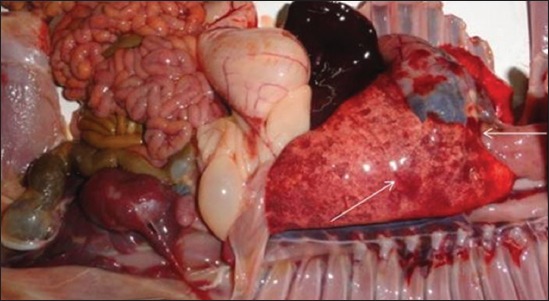
Characteristic hemorrhagic lesions on the diaphragmatic lobe of the pneumonic lung.

The clinically ill animals showed anorexia, fever (104-105°F), mucoid nasal discharge, and coughing for up to two days. A few animals showed mild diarrhea with semi-solid fecal consistency. Of the 14 sick goats, four goats died after an episode of fever and respiratory signs. On necropsy of the dead goats, pneumonia was diagnosed among all the cases and classified as acute fibrinous bronchopneumonia characterized by serofibrinous exudation in the bronchiolar lumen. The pneumonic lung tissues showed congestion and petechial and ecchymotic type of hemorrhages (Figure-1). Consolidation was recorded in whole of the apical and parts of the diaphragmatic lobes. The tracheal rings were hemorrhagic and the tracheal lumen was filled with the mucus and froth. Bacterial culture revealed isolation of Gram-negative coccobacilli and small bacilli suspected to *P. multocida* and/or *M. haemolytica*. The isolates were presumptively identified by cultural and biochemical characters. The small, smooth, white-creamy, mucoid, and hemolytic, as well as non-hemolytic colonies, were developed on the blood agar. The hemolytic colonies were further streaked on the MacConkey agar which showed a very mild growth after 48 h. The non-hemolytic colonies failed to grow on the MacConkey agar. All the isolates did not show any growth on the Deoxycholate citrate agar (HiMedia, Mumbai). *P. multocida* suspected isolates revealed Gram-negative small coccobacilli by Gram’s staining. However, the colonies suspected to *M. haemolytica* showed Gram-negative, pleomorphic, coccobacillary-to-small bacillary morphology. All the isolates were positive for the production of catalase and oxidase. The indole was produced by *P. multocida* isolates. Bacterial isolation showed the recovery of *M. haemolytica* (5) and *P. multocida* (3) of eight clinical cases. Two clinical cases were found to be complicated by both *M. haemolytica* and *P. multocida*. Moreover, *M. haemolytica* and *P. multocida* were coisolated from two of the lung tissues. Furthermore, one of the other two lung tissues showed the isolation of *M. haemolytica* while the other showed recovery of *P. multocida*. The heart blood of the dead animals showed bacterial isolation similar to the respective lung tissues. Thus, a total of 18 distinct isolates including *M. haemolytica* (n=10) and *P. multocida* (n=08) were recovered from all 14 cases.

The isolates showed susceptibility to tetracycline and chlortetracycline. However, an intermediate sensitivity was recorded against amikacin (33.3%), gentamicin (22.2%), amoxicillin (16.6%), cefotaxime (16.6%), ciprofloxacin (16.6%), and enrofloxacin (16.6%).

**Figure-2:**
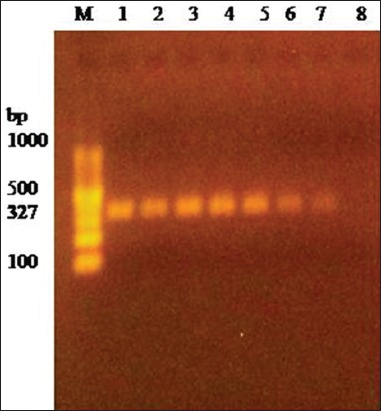
Amplification of *PHSSA* gene (327 bp) of *Mannheimia haemolytica*. Lane 1-7: Test positive. Lane 8: Test negative. Lane M: 100 bp DNA Ladder.

**Figure-3:**
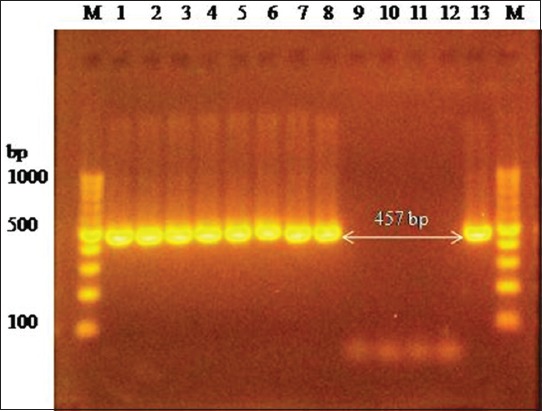
Amplification of *KMT1* gene (457 bp) specific to *Pasteurella multocida*. Lane 1-8: Test positive. Lane 9-11: Test negative. Lane 12: Negative control. Lane 13: Positive control. Lane M: 100 bp DNA Ladder.

**Figure-4:**
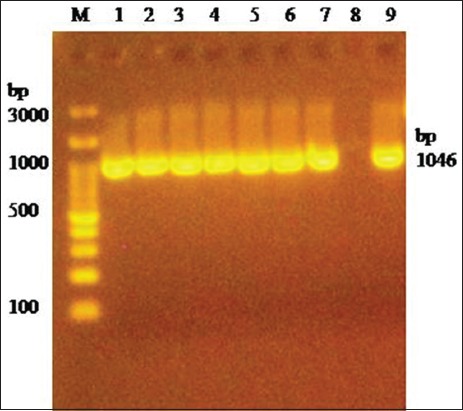
Amplification of *hyaD-hyaC* gene (1046 bp) specific to *Pasteurella multocida* serogroup A capsule. Lane 1-7: Test positive. Lane 8: Negative control. Lane 9: Positive control. Lane M: 100 bp DNA Ladder.

PCR amplification of 16S rRNA using universal primers followed by sequencing confirmed *M. haemolytica* and *P. multocida* bacterial isolates. *M. haemolytica* and *P. multocida* isolates were further identified by specific amplification of *PHSSA* (327 bp) (Figure-2) and *KMT1* (457 bp) (Figure-3) gene, respectively. Capsule typing of *P. multocida* isolates showed the presence of *hyaD-hyaC* gene specific to serogroup A (1046 bp) (Figure-4). Nucleotide sequences have been submitted to NCBI GenBank database with their respective accession numbers (MH068780.1, MH068781.1, MH068782.1, MH068783.1, and MK295774.1).

## Discussion

Globally, the goat is reared as a food animal which provides meat, leather, milk, and manure and inhabited mainly to semi-arid and arid climate. Goat farming plays an important role for sustaining the livelihood of various rural families in Chhattisgarh, India. However, variable environmental factors predispose the goats to the development of respiratory diseases including pneumonia. Pneumonic pasteurellosis is important to sheep and goats throughout the world and can be acute with fever, listlessness, dyspnea, poor appetite, and sudden death [16]. Physical environmental stresses predispose the goats to respiratory infections by means of lowering their innate resistance to *P. haemolytica* infection [16]. There is a direct correlation of sudden environmental changes in terms of rainfall, wind, temperature etc. to the occurrence, and development of pneumonia in small ruminants [17]. A confluence of important factors such as production stress in prevailing environmental conditions in a particular geographical area and changing weather patterns predispose these animals to pasteurellosis (mannheimiosis) [18]. A survey by 126 member countries of the World Organization for Animal Health (OIE) remarked that pasteurellosis has emerged as one of the major animal diseases affected by climate change [19].

The necropsy-based gross pathological observations are important to diagnose the diseases like pneumonia among animals in rural areas. Such a diagnosis of infectious diseases is very important for record keeping and National Disease Reporting System. Necropsy-based observation of fibrinous bronchopneumonia with congestion and hemorrhages of the lungs and trachea were similar to the earlier studies [20]. Among pneumonic lungs, the consolidation was recorded in whole of the apical lobe [21]. The presence of hemorrhagic lesions on the different organs, namely heart epicardium, spleen, and kidneys indicated septicemia due to these bacteria.

Pneumonia in small ruminants is primarily caused by parainfluenza-3 virus and respiratory syncytial virus and mycoplasma infection [22] and is predisposed by variable weather conditions. Respiratory viral infections affect mucociliary clearance mechanisms in lungs for removing the pathogens that reach the lower respiratory tract and thus increase the susceptibility of sheep and goats to secondary bacterial infections [16]. The respiratory viral agents create a favorable environment in the lungs supporting the bacterial growth by interfering with the mucociliary clearance mechanism of the respiratory tract and by downregulating the phagocytosis by the pulmonary macrophages [23]. However, *M. haemolytica*, the most frequently isolated bacterial pathogen from the lungs, is considered as the main cause of the pneumonic pasteurellosis/respiratory mannheimiosis [6]. Further, the respiratory syncytial virus and parainfluenza-3 virus can cause non-fatal pneumonia but are not necessary predisposing agents for *M. haemolytica*-caused pneumonia of bighorn sheep [24]. However, *M. ovipneumoniae*, a cause of non-fatal pneumonia in bighorn sheep, can predispose them to fatal pneumonia due to *M. haemolytica* infection [22]. The viral and mycoplasma investigation was not done in the present study. *M. haemolytica* significantly enhances the colonization of *P. multocida*, leading to more severe disease [7].

The isolates were susceptible to tetracycline and chlortetracycline, which might probably be due to the controlled use of these antimicrobials in the flock. Susceptibility results in the present study were more or less similar to the previous studies [25,26]. Reduced susceptibility to β-lactams (amoxicillin and cefotaxime), aminoglycosides (amikacin and gentamicin), and fluoroquinolones (ciprofloxacin and enrofloxacin) might be due to their selective use in the flock.

The presence of the virulent strains of *M. haemolytica* and/or *P. multocida* indicated their role for the pathogenesis and development of pneumonia in goats. The *PHSSA* is homologous to virulence-associated genomic fragments of *M. haemolytica*, and it could have significant pathobiological effect in the progression of pneumonic pasteurellosis [27]. *P. multocida* Type A involvement to pneumonic sheep has been reported from Tamil Nadu, India [28], and thus supported the findings of the present study. *P. multocida* lipopolysaccharide capsule plays an important role in the pathogenesis of the disease. It stimulates humoral immunity and is considered as a protective antigen [29]. It is observed that the capsule plays a significant role in the resistance to phagocytosis [30] and *P. multocida* requires a complete lipopolysaccharide structure to replicate *in vivo* and to establish the disease.

Isolation and identification of *M. haemolytica* and *P. multocida* based on the cultural and biochemical characters and by the PCR assays targeting the specific genes have been reported earlier similar to the present study [13,31]. Moreover, the specific identification of *P. multocida* by 16S rRNA and *KMT1* gene and the characterization by capsular typing has a significant impact on the epidemiology and control of pasteurellosis in domestic animals [8,32,33]. It is also proposed that the molecular typing methods provide rapid bacterial identification and have proved to be more specific over the cultural and biochemical tests [13,34].

## Conclusion

High prevalence of pneumonic pasteurellosis that leads to high goat mortality was recorded in the backyard flock of a village in Chhattisgarh. The occurrence of goat pneumonia might have been predisposed due to environmental and nutritional stress. Acute fibrinous bronchopneumonia was predominantly recorded among the dead goats. Isolation and molecular characterization of *M. haemolytica* and *P. multocida* confirmed the occurrence of pneumonic pasteurellosis or respiratory mannheimiosis in the affected goats.

## Authors’ Contributions

NR was involved in the disease investigation in the field, planning, sampling, and bacterial isolation; VRG, FFK, KKK, and RKS performed sample processing and microbiological analysis; DDH and DKJ performed molecular analysis and manuscript writing. All authors read and approved the final manuscript.
